# Supplementary material for: Gene-Based Resistance to Erysiphe Species Causing Powdery Mildew Disease in Peas (Pisum sativum L.)
Source: Genes (Basel). 2022 Feb 8;13(2):316. doi: 10.3390/genes13020316 (PMC8872628; doi:10.3390/genes13020316)
Supplement: Supplementary file 1 [file genes-13-00316-s001.zip › genes-1416396-supplementary.pdf]

Table S1. Functional Markers reportedly linked to different *er1* alleles. 1

Gene Variation	Marker Name	Type	Primer Sequence (5'-3')	Reference
er1-1	*er1-1/AsuHPI-B*	CAPS	**Fwd**-AGGTTTGCAAGGGACACAAC<br>**Rev**-TGAAGAAGCTAACCTGATTCAACC	[168]
er1-1	KASPar-*er1-1*	KASPar	**Fwd-C:** CCCTTACAATCCATAACAAAATAGGTG<br>**Fwd-G:** CCCTTACAATCCATAACAAAATAGGTC<br>**Common Rev:** TTTGCAAGGGACACAACATTTGGAAGAA	[107]
er1-2	*er1-2*/MGB	STS	**Fwd**-CCAAAGGAGGGAAAGGAAAC<br>**Rev**-GGAGCAGGTGACAGGAGAC	[168]
er1-3	*er1-3*/XbaI	dCAPS	**Fwd**-CAATTGAAGAGGATTTTAAAGTTGTTCTAG<br>**Rev**-GCCAGATAGTTGGACTGCAAG	[168]
er1-3	KASPar-*er1-3*	KASPar	**Fwd-G:** GTATATTTAATCTTAAGTCACACCTTATTCC<br>**Fwd-N/A:** AGTATATTTAATCTTAAGTCACACCTTATTCT<br>**Common Rev:** AGATCAATTGAAGAGGATTTTAAAGTTGTT	[107]
er1-4	CAPS	*er1-4*/AgsI	**Fwd**-GCTGTTGCAGTTGTGTGTCTT<br>**Rev**-GAACAAGGATGCCAAGTTGA	[168]
er1-4	KASPar-*er1-4*	KASPar	**Fwd-A:** GTGTCTTGTGTTGCTAGCTGTTTCAA<br>**Fwd-N/A:** GTGTCTTGTGTTGCTAGCTGTTTCAT<br>**Common Rev:** TAGAACGAACCATGCTTAGCTTACCTTT	[107]
er1-5	*er1-5*/HRM54	HRM	**Fwd**-GATGAGGAAGTGGAAGACTT<br>**Rev**-AATTGATATTCAACTGTTCTTGTC	[168]
er1-5	KASPar-*er1-5*	KASPar	**Fwd-G:** ATTCAACTGTTCTTGTCTCATCTTCC<br>**Fwd-A:** GATATTCAACTGTTCTTGTCTCATCTTCT<br>**Common Rev:** TTTCTTCAGATGAGGAAGTGGAAGACTT	[107]
er1-5	GIM-300/SmlI	CAPS	**Fwd**-TCTGCATATGGAATTCACCAA<br>**Rev**-AATTGATATTCAACTGTTCTTGTC	[96]
er1-6	KASPar-*er1-6*	KASPar	**Fwd-T:** TTGAAGTTACCTGAAAGAGAACAA<br>**Fwd-C:** CTTTGAAGTTACCTGAAAGAGAACAG<br>**Common Rev:** GTCCTCACCTTCTTCTCTTCACGAT	[107]
er1-7	InDel111–120	indel	**Fwd**-GGAGTTAAGGAACGAACTTTGG<br>**Rev**-CCATGTCTGCGTCTGTATCTTT	[69]
er1-7	KASPar-*er1-7*	KASPar	**Fwd-TCATGTTATT:** AGCTGTTTCAATCTTAATTGAACATATTATT<br>**Fwd-N/A:** AGCTGTTTCAATCTTAATTGAACATATTATG<br>**Common reverse:** ATAGAACGAACCATGCTTAGCTTACCTTT	[107]
er1-8	*InDel-er1-8*	indel	**Fwd**- GTTTTGACTGATATGACAGATGGGA<br>**Rev**-GTTTGTAGACTGTCGCTGTTTCC	[102]

er1-8	KASPar-<br>*er1-8*	KASPar	**Fwd-TGG:** TGGCAACAGCGCTTAAGAACTGG<br>**Fwd-** GAGCAACAGCGCTTAAGAACTGG<br>**Common Rev:**<br>TGGTTGGTTTCATGGTTGATCCCATC	[102]
er1-9	*KASPar-*<br>*er1-9*	KASPar	**Fwd T:** TTTTGTTATATGGGCAGGGTGGTATT<br>**Fwd**-TGTTATATGGGCAGGGTGGTATC<br>**Common Rev:**<br>CAAAATGTAGATTATGCTTACAATTAGTGGA	[102]
er1-10	KASPar-<br>*er1-10*	KASPar	**Fwd-G:** TACAATTAGTGGAAGAAATGGAAGC<br>**Fwd-A:**<br>GCTTACAATTAGTGGAAGAAATGGAAGT<br>**Common Rev:**<br>GTTATATGGGCAGGGTGGTATTCTTATTA	[107]
er1-11	KASPar-<br>*er1-11*	KASPar	**Fwd-N/A:** ATGCAAATCTCATGCGCGTGTGTA<br>**Fwd-GA:** GCAAATCTCATGCGCGTGTGTG<br>**Common Rev:**<br>TCAGGATTCAAGATGAGATTCATGTACAAA	[107]
